# Multidisciplinary Collaboration between a Community Pharmacy and a Travel Clinic in a Swiss University Primary Care and Public Health Centre

**DOI:** 10.3390/pharmacy6040126

**Published:** 2018-12-05

**Authors:** Jérôme Berger, Marie-José Barbalat, Vanessa Pavón Clément, Blaise Genton, Olivier Bugnon

**Affiliations:** 1 Community Pharmacy Centre, Department of Ambulatory Care and Community Medicine, University of Lausanne, CH-1011 Lausanne, Switzerland; marie-jose.barbalat@hospvd.ch (M.-J.B.); vanessa.pavon-clement@hospvd.ch (V.P.C.); olivier.bugnon@hospvd.ch; (O.B.); 2 Community Pharmacy Practice Research, School of pharmaceutical Sciences, University of Geneva/University of Lausanne, CH-1206 Genève, Switzerland; 3 Travel Clinic, Department of Ambulatory Care and Community Medicine, University of Lausanne, CH-1011 Lausanne, Switzerland; blaise.genton@chuv.ch; 4 Infectious Disease Service, University Hospital, CH-1011 Lausanne, Switzerland; 5 Swiss Tropical and Public Health Institute, CH-4051 Basel, Switzerland

**Keywords:** travel medicine, pharmacy, community, travel, practice, vaccination, Switzerland, multidisciplinary collaboration

## Abstract

This review is a narrative description of a collaboration between a travel clinic and a community pharmacy centre within a university primary care and public health centre (Lausanne/Switzerland). Pharmacists and pharmacy technicians participate in this collaboration to provide (1). counselling and clinical activities with travellers (e.g., pre-travel consultations and advice to travellers), (2). clinical pharmacy expertise and medicine information services (e.g., selection of an appropriate antimalarial medication for a traveller to manage of drug-drug interactions), (3). technical and logistical activities related to medicines and vaccines (e.g., management of vaccine shortages and specially imported medicines and vaccines from foreign countries) and (4). educational activities (e.g., undergraduate pharmacy teaching and continuous education to community pharmacists). Such a multidisciplinary collaboration should be encouraged as it enables us to address the evolution and challenges of travel medicine related to medication, such as growing vaccine shortages and an increasing number of chronic patients who travel. This review may be used as a model for the dissemination of such collaborative practices, to develop future advanced teaching and training activities, to provide a framework for research related to travel and medicines and to participate in the evaluation of vaccination practices by community pharmacists.

## 1. Introduction

The Department of Ambulatory Care and Community Medicine of Lausanne (in the French-speaking part of Switzerland) is a university primary care and public health centre with health care workers that include general practitioners, nurse practitioners, and community pharmacists. Almost 600 people work in this centre, including 145 physicians and 172 other health care professionals (such as nurses and community pharmacists). Next to its community pharmacy centre (which includes a community pharmacy as well as a research unit), the centre hosts a travel clinic, where physicians, nurses, pharmacists and pharmacy technicians care for adult, geriatric and child travellers. The head of the community pharmacy centre is a professor of pharmacy practice and the head of the travel clinic is a professor of tropical and travel medicine. This academic setting facilitates the development of collaborative practice and of research and teaching activities.

The travel clinic is one of the two major centres in the French-speaking part of Switzerland. In 2017, the clinic administered 3900 yellow fever vaccines and performed more than 11,000 pre-travel consultations. The Swiss mandatory health insurance does not reimburse patients for pre-travel consultations. Hence, to ensure an affordable service, the aim of the travel clinic is to provide a standard pre-travel consultation in approximately 20 min. Post-travel consultations (e.g., for a traveller who is referred by the emergency department concerning a health problem after returning from a trip abroad) are also performed in the clinic. The characteristics of the travellers who are consulted in this travel clinic have already been described elsewhere [1]. In brief, the mean age of travellers is 32 years. Pre-travel consultations are sought approximately one month before departure (median of 29 days). Forty-six percent of travellers had at least one pre-existing medical condition (e.g., 9.4% reported a psychological or psychiatric problem, 1.8% a cancer and 0.4% an HIV infection). Most of them were travelling to Africa (46%), followed by Asia (35%) and Latin America (20%). Tourism (75%) and visits to friends and relatives (18%) were the main reported reasons to travel. At least one vaccine was administered to 99% of travellers [1].

The aim of this review is to present the multidisciplinary activities that occur within the travel clinic among physicians, nurses, community pharmacists and pharmacy technicians, as well as the educational activities of pharmacists related to travel medicine and vaccines.

## 2. Materials and Methods

To conduct this review, data were collected regarding the training of pharmacists involved in clinical activities with travellers, the activities and services of pharmacists and pharmacy-technicians related to the travel clinic and the educational activities of the pharmacy centre related to travel medicine and vaccines. Data assessment was based on the review of: 1. the 2017 annual report of the Department of Ambulatory Care and Community Medicine of Lausanne that summarizes clinical, research and educational activities; 2. the 2017 statistical directory of the Department of Ambulatory Care and Community Medicine of Lausanne that provides the main management indicators (e.g., number of patients, staff resources and budgets); 3. data extracted from custom-made software (DIAMM/G) that records the clinical activities in the travel clinic and 4. data extracted from custom-made administrative software (Allegro) used for human resources management by the Department of Ambulatory Care and Community Medicine of Lausanne. The findings are presented as a narrative description.

## 3. Results and Discussion

The main activities (summarized in Table 1) include counselling and clinical activities with travellers, providing clinical pharmacy expertise and medicines information services, technical and logistical activities related to medicines and vaccines and education activities.

### 3.1. Counselling and Clinical Activities with Travellers

Since 2009, pharmacists have been involved in the counselling and clinical activities of the travel clinic. As nurses and physicians consulting in the travel clinic, the pharmacists give advice according to travel purposes and patterns and the medical conditions of travellers, perform vaccinations (e.g., vaccinations for yellow fever, typhoid, rabies, and others), prescribe antimalarial medicines (for prophylaxis or stand-by emergency treatment) and collect blood samples for serology.

Currently, two pharmacists perform regular activities in the travel clinic. In 2017, they managed 137 and 166 pre-travel consultations, respectively. These pharmacists have been specifically trained to join the travel clinic. Their training was certified by a “Certificate in Travel Health” (International Society of Travel Medicine), a “Certificate of Achievement of Pharmacy-Based Immunization Delivery” (American Pharmacist Association) and a (in French) “Certificat de formation complémentaire FPH Vaccination et prélèvements sanguins (Foederatio Pharmaceutica Helvetiae)” [2]. The latter is a Swiss post-graduate training certificate that is mandatory for pharmacists who administer vaccines and it demonstrates skills in vaccination, injections and blood sample collection and processing techniques. In Switzerland, pharmacists who perform vaccinations must follow a mandatory training day every for two years to renew their “Certificat de formation complémentaire FPH Vaccination et prélèvements sanguins”. In addition to this mandatory education, both pharmacists also completed continuing education on vaccines and travel medicine by participating in the monthly seminar held by the travel clinic and by attending the “Swiss Tropical and Public Health Institute - International Short Course on Travellers’ Health”, the (in French) “Congrès Suisse de Vaccination” and the (in French) “Journée Romande de Médecine des Voyages”.

Under the supervision of pharmacists, pharmacy technicians take part in the travel clinic activities. The technicians charge the amounts that are linked to pre-travel consultations. This administrative task gives them the opportunity to complete the prevention and care advice for the travellers, mainly on the responsible use of antimalarial medicines (e.g., management of missed doses), safe sex, sun protection, mosquito bite prevention, water purification and treatment for motion sickness. If needed, technicians can supply travellers with medicines prescribed during the pre-travel consultations (e.g., antimalarials, antibiotics, preventive or acute treatment of altitude sickness) and they can deliver non-prescription medicines (e.g., antiemetics or antidiarrheals). Various items, such as sun protections, water filters, tablets for water disinfection, and mosquito nets or repellents, are actively promoted if needed. In 2017, pharmacy technicians delivered medicines, sanitary materials and/or pharmacy kits to approximately 3600 travellers after their pre-travel consultation at the travel clinic. Travellers are also free to buy medicines and other items in their usual community pharmacy.

### 3.2. Clinical Pharmacy Expertise and Medicines Information Services

In addition to the two pharmacists providing consultations in the travel clinic, other pharmacists also support the travel clinic activities. A medicines information service (MIS), run by pharmacists, is available from Monday to Saturday. The MIS offers a helpline related to medication management for health care professionals and patients and it manages the access to several medicine databases and to specific resources related to vaccines or travel medicine. The MIS also develops and updates practice recommendations and databases to promote the responsible use of medicines, such as antimalarials or vaccines. Such tools and databases include a drug-drug interaction database specifically dedicated to antimalarials, a database on temperature excursion management and several medicine comparison charts related to antimalarials, anti-infectives, vaccines and medications used in the prevention of deep vein thrombosis. The MIS also publishes patient information leaflets in French related to the use of anti-infectives that are not marketed in Switzerland but that may be prescribed by the travel clinic. Such medicines are imported from foreign countries (see below), and their packages do not include a patient leaflet in French. These medicines include diethylcarbamazine, niclosamid, praziquantel or primaquine. In addition, the MIS publishes patient information leaflets on travelling with medicines and supports the provision of certificates for carrying medicines (including narcotics).

In 2017, the MIS answered approximately 1800 phone requests, mainly from health care professionals within the primary care and public health centre. Among these, 804 questions were raised by the travel clinic. Questions from the travel clinic were mostly asked by nurses (81%), followed by physicians (12%). The majority (80%) concerned drug-drug interactions with antimalarials, either as chemoprophylaxis or as stand-by emergency treatment. Consultation with the MIS allows care providers to select the safest antimalarials, considering the individual medication plan of the travellers and according to their medical conditions (e.g., contraindications or allergies). The development and updates of internal tools and databases and the training of pharmacists mean that most of these 804 questions can be answered in a period that is compatible with pre-travel consultations: 79% of the questions were answered in less than 5 min and 17% in 6 to 15 min. One of the advantages of such a helpline is to allow clinicians to focus on the pre-travel consultation, while a pharmacist works in parallel to check for potential drug-drug interactions and looks for the best alternatives, if necessary, allowing the consultations to be shortened.

### 3.3. Technical and Logistical Activities Related to Medicines and Vaccines

In addition to consultation activities, the community pharmacy team ensures good provision and distribution of medicines and vaccines that are administered at the travel clinic or delivered to travellers. The supply chain of medicines and vaccines is ensured by the pharmacy, from orders to the stock management within the consulting rooms and fridges, including the proper disposal of unused medicines and vaccines. Such activity includes a continuous control by pharmacy technicians of the temperature of the cooling chamber and refrigerators used to stock vaccines in the travel clinic. In the case of an alarm (i.e., if the recorded temperature goes below 2 °C or above 8 °C), the MIS determines the action to take based on a database related to temperature excursion management. In 2017, the pharmacy managed 17 alarms in the travel clinic.

The pharmacy team also manages medicines and vaccine shortages, which are more and more frequent [3], in collaboration with physicians from the travel clinic. For example, in the case of a vaccine shortage, the pharmacy team gathers information on the currently available stock in the pharmacy and travel clinic, the recent number of injections of the vaccine, the probable next availability of the vaccine and the potential available alternatives (e.g., to continue vaccination schemes). Based on this information, a potential restricted administration of the vaccine during the shortage is discussed (e.g., which travellers should be vaccinated, with priority depending on their medical conditions and planned trips). This information is then disseminated within the travel clinic (e.g., the remaining stock of a vaccine may be taken out of the travel clinic to the pharmacy and delivered to health care professionals on request for administration to travellers who meet pre-established medical or travel conditions). In addition, a list of information and practice recommendations are made available to the pharmacy team to answer to other community pharmacies or physicians seeking for advice on the management of vaccine shortages.

Finally, importing special medicines and vaccines that are not marketed in Switzerland is performed by the pharmacy team for the travel clinic. Based on legal requirements, the pharmacy requests required import licenses from national health authorities (Swissmedic). In Switzerland, this is mandatory for vaccines imported from foreign countries [4]. In such cases, the pharmacy requests authorization to administer a foreign vaccine and authorizations linked to the import of every batch of a vaccine. Once all authorizations have been obtained, the pharmacy coordinates the importation process with a foreign wholesaler and Swiss customs. The entire process includes completing the requested forms (including clinical reasons to import a foreign vaccine, information on the pharmaceutical company, the clinicians who are responsible for administration of the vaccine and other information), paying the due taxes and ensuring full traceability of such imported vaccines (from information on the number of vaccines and batch numbers that have been received to the identities of the patients who have been administered injections). For example, 250 doses of typhoid-injectable vaccines were specially imported in five orders from France in 2017, because no such vaccine is marketed in Switzerland. These vaccines are indicated for patients in whom an oral live typhoid vaccine is contraindicated (e.g., in the case of drug-drug interactions, contraindications or too short of a delay prior to potential typhoid exposure).

### 3.4. Education Activities

The expertise gathered during the multidisciplinary activities at the travel clinic allows the pharmacists of the Department of Ambulatory Care and Community Medicine to teach other community pharmacists, pharmacy technicians and students about vaccines and travel medicine. For more than 10 years, master’s students at the University of Geneva have been taught 4 h of education in the community pharmacy on counselling travellers (e.g., malaria prevention or travelling with medicines) and 4 h on immunization (e.g., vaccination schemes, vaccination booklets, advice on vaccinations in the community pharmacy). In the near future, injection and blood sample collection techniques will also be included in the master’s programme. Indeed, pharmacists have been authorized to administer vaccines (e.g., influenza) in Switzerland for some years (depending on the canton). These new undergraduate courses will fulfil the revised national learning objectives for pharmacists, based on the Swiss Federal Act on Medical Professions [5].

In 2017, the pharmacy team offered continuous education sessions on vaccines and travel medicine to community pharmacists and pharmacy technicians in several locations throughout the French-speaking part of Switzerland. Four seminars (lasting 1 to 2 h) were conducted on updates to vaccination schemes for community pharmacists. Four full days of training were conducted for pharmacy technicians on the management of vaccination booklets (paper and electronic versions) and advice on vaccination in community. Finally, five full days (including case studies) of training were conducted for community pharmacists on immunizations, vaccination schemes and the management of information on sources on immunizations.

## 4. Conclusions

To the best of our knowledge, this report describes a unique example of a multidisciplinary collaboration between a university travel clinic and a university community pharmacy centre, at least in Switzerland. This report describes the benefits of a collaborative practice between pharmacists, pharmacy technicians, nurses and physicians in the activities of a travel clinic. As pharmacists and pharmacy technicians take part in the counselling and clinical activities with travellers, they gain knowledge and competence related to travel medicine and vaccines. Additionally, pharmacists and pharmacy technicians can bring their specific expertise and knowledge related to the responsible use of medicines to the travel clinic.

This effort goes beyond daily interactions between colleagues or completing the delivery of prescribed medicines and immunizations with responsible self-medication. This type of comprehensive collaborative practice is an advantage for travellers and for health professionals. Indeed, this practice supports the evolution of travel medicine and allows care providers to better face the challenges of travel medicine related to medications, such as growing vaccine shortages, an increasing number of chronic patients who travel and the increasing complexity of medicines administered while travelling abroad, such as self-administered injectables. As pharmacists are authorized to perform vaccinations in several countries and in most of the Swiss cantons, collaborations with travel clinics should be encouraged. The present review could serve as a model for the dissemination of such practices. The experience gained should form the basis for further teaching and training activities, including advanced training for pharmacists who are already authorized to perform vaccinations. In addition, this narrative may provide a framework for research related to travel, vaccines and medicines (e.g., evaluation of vaccination practices by community pharmacists or assessment of medication safety of chronic patients who travel).

## Figures and Tables

**Table 1:** Main figures related to the activities of pharmacists and pharmacy-technicians within the travel clinic or related to travel medicine and vaccines (data from 2017).

Main Activities	Main Figures (From 1 January to 31 December 2017)
Counselling and clinical activities with travellers	303 pre-travel consultations managed by pharmacists Approximately 3600 travellers supplied with medicines, sanitary materials or pharmacy kits by pharmacy technicians
Clinical pharmacy expertise and medicines information services	804 questions from the travel clinic managed by pharmacists
Technical and logistical activities related to medicines and vaccines	17 temperature excursion management activities in the travel clinic
Education Activities	8 h of undergraduate pharmacy teaching (travellers’ counselling in the community pharmacy and immunization) 4 seminars (1–2 h) for community pharmacists (updates to vaccination schemes) 4 full days for pharmacy technicians (vaccination booklets and advice on vaccinations) 5 full days for pharmacists (immunization training, vaccination schemes and management of information)
